# 66 Optic neuritis in children

**DOI:** 10.1093/rheumatology/keac496.062

**Published:** 2022-10-07

**Authors:** S Melzi, R Aboura, A Ladjouze, N Bouhafs, K Boulesnane, R Bouadjar, L Mebrouki, Z Beddek, N Haddad, M Tabti, Z Bouzerar

**Affiliations:** General Pediatrics Department, CHU of Bab el oued, Algiers, Algerie

## Abstract

**Introduction:**

Optic neuritis (ON) are rare in children. They are due to inflammation of the optic nerve which results in a sudden drop in visual acuity, or amputation of the visual field. They are often secondary to an infectious disease or vaccination, but they can be part of auto-inflammatory, demyelinating or systemic diseases. The features of optic neuritis in children are different from adults as regards high rate of bilateral involvement, poor visual acuity, and papillitis.

**Aim:**

Our aim is to describe the clinical and therapeutic outcomes of patients diagnosed with optic neuritis in the general pediatrics department at the CHU of Bab el Oued in Algiers.

**Methods:**

It is a retrospective, descriptive study of patients who were treated for optic neuritis in our hospital over a period of three years (January 2018 to April 2021).

**Results:**

There were 7 cases, 4 girls and 3 boys with F: M of 1.3:1, mean age of 10 years and age range of 6–13 years. The first manifestation was a decrease in visual acuity in all cases, 12 eyes affected, with bilateral involvement in 5 patients. Visual acuity decrease was severe < 3/10 in 80% of cases. The visual field was pathological in 2 cases (narrow, central scotoma). All patients had headaches.

Retrobulbar involvement was confirmed by visual evoked potentials in all patients, they showed demyelinating involvement. Three patients presented papillitis.

The diagnostic work-up included cerebral imaging which showed old nodular abnormalities of signal of the subcortical white matter in one patient.

CSF analysis revealed anti-MOG antibodies in only one case. The infection investigations were negative in all cases except positive COVID 19 serology in one case

The diagnosis retained in our patients were 2 cases of Behcet's disease, 1 monophasic neuritis, 1 optic neuritis with anti-MOG, 1 CRION, 1 case linked to COVID 19 in the absence of another plausible cause. In one case, no diagnosis was retained.

Seventy percent of the patients benefited from treatment with corticosteroids pulse, only 2 did not receive treatment, the short-term evolution was favorable in 5 patients with a total recovery of visual acuity, one patient has evolved into atrophy of the left optic nerve and kept visual acuity low to 1/10 of the left eye.

The medium-term evolution was marked by recurrence in 3 patients, 5 episodes in the 2 patients with Behcet's disease and 8 episodes in CRION which required the use of immunosuppressive treatment and biotherapy for Behcet's disease and plasmapheresis for CRION.

**Conclusion:**

Despite the fact that our small series of patients is not representative, we found similar characteristics described in children in other studies. The average onset age was 10 years, with a female predominance, the severe decrease in visual acuity with frequent bilateral involvement. The diagnosis of ON is clinical and must be considered in any sudden drop in visual acuity. The diagnosis of the underlying pathology is often difficult but it’s important in order to propose an adequate specific treatment. The treatment of children's ON is controversial and usually extrapolated from adults. Most practitioners use corticosteroids pulse. The visual prognosis seems good in children, particularly in the case of bilateral involvement, but depends essentially on the aetiology.

